# A distributed integral control mechanism for regulation of cholesterol concentration in the human retina

**DOI:** 10.1098/rsos.240432

**Published:** 2024-10-30

**Authors:** Ronél Scheepers, Noa L. Levi, Robyn P. Araujo

**Affiliations:** ^1^ School of Mathematical Sciences, Queensland University of Technology (QUT), Brisbane 4000, Australia; ^2^ School of Mathematics and Statistics, University of Melbourne, Victoria 3010, Australia

**Keywords:** robust perfect adaptation, chemical reaction networks, antithetic integral control, retinal cholesterol homeostasis

## Abstract

Tight homeostatic control of cholesterol concentration within the complex tissue microenvironment of the retina is the hallmark of a healthy eye. By contrast, dysregulation of biochemical mechanisms governing retinal cholesterol homeostasis likely contributes to the aetiology and progression of age-related macular degeneration (AMD). While the signalling mechanisms maintaining cellular cholesterol homeostasis are well-studied, a systems-level description of molecular interactions regulating cholesterol balance within the human retina remains elusive. Here, we provide a comprehensive overview of all currently-known molecular-level interactions involved in cholesterol regulation across the major compartments of the human retina, encompassing the retinal pigment epithelium (RPE), photoreceptor cell layer, Müller cell layer and Bruch’s membrane. We develop a comprehensive chemical reaction network (CRN) of these interactions, involving 71 molecular species, partitioned into 10 independent subnetworks. These subnetworks collectively ensure robust homeostasis of 14 forms of cholesterol across distinct retinal cellular compartments. We provide mathematical evidence that three independent antithetic integral feedback controllers tightly regulate ER cholesterol in retinal cells, with additional independent mechanisms extending this regulation to other forms of cholesterol throughout the retina. Our novel mathematical model of retinal cholesterol regulation provides a framework for understanding the mechanisms of cholesterol dysregulation in diseased eyes and for exploring potential therapeutic strategies.

## 1. Introduction

**Figure 1.**
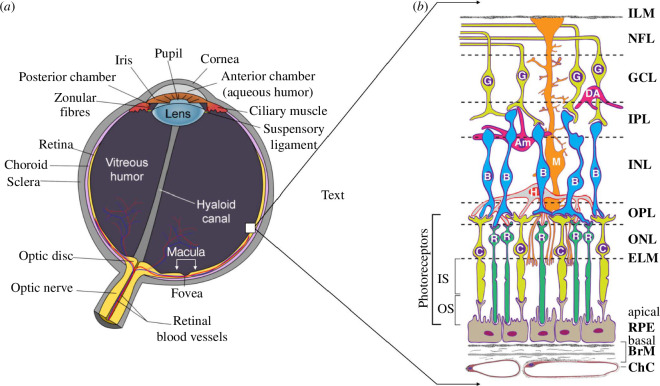
Structure of the retina. (*a*) Schematic cross-section of the human eye. The neural retina and choriocappilaris are part of the inner lining, shown in yellow. The macula and fovea (a depression in the macula) are bracketed in white. (*b*) Chorioretinal cells and layers. Cells: RPE, retinal pigment epithelium; C, cone photoreceptor; R, rod photoreceptor; H, horizontal cell; B, bipolar cell; M, Müller glial cell; Am, amacrine cell; DA, displaced amacrine cell; G, ganglion cell. The area between the foot processes of the Müller cell and the RPE layer forms the subretinal space [2]. Layers: ChC, choriocapillaris; BrM, Bruch’s membrane; ELM, external limiting membrane; ONL, outer nuclear layer; OPL, outer plexiform layer (synapses); INL, inner nuclear layer; IPL, inner plexiform layer; GCL, ganglion cell layer; NFL, nerve fibre layer; ILM, inner limiting membrane. This image is a derivative of ‘Anatomy of the eye’ [3] and ‘Human eye’, taken from [4].

**Figure 2.**
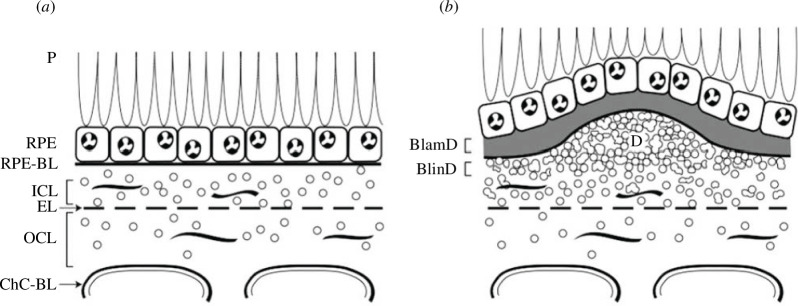
Healthy RPE/BrM versus AMD lesions. Sectional views of the RPE/BrM complex from a healthy eye (*a*), compared with an eye with AMD (*b*). Small circles represent EC-rich/ApoB lipoproteins, native, and coalesced into drusen (D). (*a*): P, photoreceptors; RPE, retinal pigment epithelium; RPE-BL, RPE basal lamina; ICL, inner collagenous layer; EL, elastic layer; OCL, outer collagenous layer; ChC-Bl, basal lamina of choriocapillary endothelium. (*b*): BlamD, basal laminar deposit; BlinD, basal linear deposit; D, drusen. (Modified from [5].)

In the healthy human eye, vision is accomplished through phototransduction, where photoreceptors in the neural retina receive and transmit visual stimuli to the brain for subsequent processing [1]. The neural retina is a complex sensory tissue found in the posterior part of the eye and lies above a single layer of post-mitotic cuboid cells called the retinal pigment epithelium (RPE) (figure 1). The RPE’s apical membrane interacts closely with photoreceptor outer segments via long microvilli, while its basolateral membrane faces Bruch’s membrane (BM), which separates it from the choriocapillaris endothelium. BM consists of five layers; the basal lamina of the RPE, inner collagenous layer (ICL), elastic layer (EL), outer collagenous layer (OCL) and the basal lamina of the choriocapillary endothelium (BL) (figure 2). Tight junctions between the RPE cells play a crucial role in establishing its apical-to-basolateral polarity and in forming the outer retinal-blood barrier to prevent diffusion of large molecules from the choroid to the retina [6,7]. Starting from the RPE’s embryonic origin, coordinated maturation requires the cells to adapt to different functional properties of the retina. For example, RPE cells in the macula (the 6 mm diameter region in the centre of the retina responsible for central vision) are smaller compared with peripheral RPE cells, have a higher melanin content and are associated with a higher number of photoreceptor cells per RPE cell than in the periphery [8].

In the diseased state of age-related macular degeneration (AMD), central, acute vision is impaired or lost. Both types of AMD, neovascular AMD (nAMD) and geographic atrophy (GA), share hallmark pathological features—soft drusen and basal linear deposit (BLinD)—constituting an oil spill on Bruch’s membrane (BM) [9]. Soft drusen and BLinD are two forms of the same extracellular lipid-rich material. More specifically, the soft drusen/BLinD deposit forms in the sub-retinal pigment epithelium-basal lamina (subRPE-BL) space, an area between the RPE-BL and the inner collagenous layer of BM (see figure 2*b*) [10].

By contrast, pre-BLinD is a layer of three to four rows of densely packed, 60−80 nm lipoprotein particles observable in many healthy older eyes, in the same space, and first called the lipid wall [9–11]. Interestingly, it has been shown that these lipoprotein particles first gather in the outer collageonous layer (OCL) of BM in early childhood, and as age increases, these particles gradually fill the subRPE-BL space to form the lipid wall [12]. While various hypotheses exist, it is not clearly understood what age-related changes predispose the progression from constitutively produced individual particles to the pathological state of soft drusen/BLinD development in some individuals.

Significantly, histochemical, biochemical and ultrastructural methods confirmed the presence of unesterified cholesterol and esterified cholesterol in both pre-BLinD and BLinD deposits [13–16]. Looking upstream, it has been proposed that these lipoprotein particles are co-translationally assembled in the RPE and deposited into the subRPE-BL space as a way to remove any excess lipids originating from multiple cellular cholesterol metabolism pathways. In particular, excess cholesterol could stem from exogenous uptake of low-density lipoproteins (LDL), high-density lipoproteins (HDL), biosynthesis of cholesterol in the RPE and phagocytosed photoreceptor (PR) outer segment (OS) tips [9,10].

Homeostasis, also known as robust adaptation in biology, refers to the coordinated physiological processes by which biological systems maintain the fixed and stable internal conditions required for survival, in the face of a wide range of possible disturbances. Retinal cholesterol homeostasis entails the interplay between *de novo* synthesis, uptake, sterol transport, metabolism and efflux within and between the four main cellular layers of the retina, the choriocapillaris (CC), Bruch’s membrane (BM), the retinal pigment epithelium (RPE) and the light-sensitive cells, also referred to as photoreceptor cells (PR). Defects in these complex processes are associated with several congenital and age-related disorders of the visual system [10,17]. Considering cholesterol’s presence in the pre-BlinD and BLinD deposits in BM, a potential link between the homeostatic state of cholesterol concentration in the healthy cell and the consequence of pathology due to disrupted homeostasis is worth investigating.

The fundamental processes in cholesterol metabolism in the vertebrate retina have been studied extensively, particularly under normal physiological conditions. However, how these individual processes are integrated into signalling biochemical networks at the intracellular and intercellular levels to achieve cholesterol homeostasis at the tissue level in the retina is currently unknown. It is only when the numerous signal transduction processes involved in cholesterol regulation are comprehended that we can explore and clarify how disruptions in these signalling pathways may lead to pathological conditions.

The homeostatic or adaptive property plays a vital role in biochemical reaction networks, and how a system adapts to changes can be described as either robust adaptation or robust perfect adaptation (RPA). Robustness refers to a system’s ability to maintain its functions and performance despite disturbances to its environment, or alterations in system parameters. Robust adaptation of a molecular concentration occurs when the concentration reacts to a perturbation and then returns at steady-state to a level close to its pre-perturbation value. RPA is a mathematical idealization of this behaviour, where the steady-state concentration returns exactly to its pre-perturbation value, regardless of the magnitude of the disturbance and independently of special parameter choices [18–20]. A molecule is thus considered ‘RPA-capable’ when its steady-state value is always constrained to adopt some fixed ‘setpoint’. RPA is thus a particular and stringent form of homeostasis, that lends itself to rigorous mathematical analysis. Moreover, RPA is typically considered in terms of its presence or absence rather than being quantified (in terms of the specific value of the invariant setpoint), and is thus strictly a network property, or ‘structural’ property, of the system. It is now known that RPA requires a modular network design, comprising one or more of two possible modules—‘opposer’ modules (with an overarching feedback structure), and ‘balancer’ modules (with an overarching feedforward structure) [18,19].

In our recent work [21], we have shown that the homeostasis imposed on subcellular cholesterol concentrations in the mammalian cell indeed corresponds to the rigorous form of regulation known as RPA. Specifically, we showed that robust homeostasis is achieved through endogenous antithetic integral feedback control, an example of a specific class of molecular control strategies known as maxRPA [22]. Hereafter, all instances of this type of controller structure are called antithetic integral control. Having suggested foundational molecular mechanisms imposing cholesterol homeostasis in mammalian cells in general, we seek here to extend our model to investigate the specific molecular mechanisms involved in maintaining cholesterol concentration in the mammalian retina.

Here we extend our prior work on cellular-level cholesterol regulation to a novel and comprehensive tissue-level description of cholesterol regulation in the human retina. This new framework encompasses all major tissue compartments in the retina, including the RPE, the PR and Müller glial cells, and provides a mechanistic connection between cellular-level regulatory mechanisms and intercompartmental tissue-level biochemical mechanisms for maintaining tightly regulated concentrations of pre-BLinD lipoprotein particles. Furthermore, our modelling results are consistent with recently suggested mechanisms by which RPE cells limit cholesterol input from the systemic circulation to stimulate cholesterol biosynthesis in the neural retina (NR), as postulated by El-Darzi *et al*. [7].

In §2, we comprehensively review the known and theorized molecular interactions and reactions in regulating cholesterol in the four-layer retina. This section delves into the intricate control of cholesterol homeostasis within the retina. We discuss critical signalling pathways at the transcriptional, translational and post-translational levels, highlighting how these processes vary across different subcellular compartments and organelles within the retinal layers. Interested readers are referred to an extensive review of these signalling processes in our recent work here [21].

We translate the detailed molecular interactions into a chemical reaction network (CRN) in §3 to allow us to generate a system of rate equations that can be analysed computationally. Section 4 is dedicated to a graphical and polynomial analysis of the retinal cholesterol CRN to identify which molecules are directly regulated by an RPA-conferring control mechanism, the species that inherit RPA within the CRN and the steady state set-points of these RPA-capable species.

## 2. The role of cholesterol in the retina

In the human retina, two types of photoreceptors, rods and cones, function in the phototransduction process, the initial step of vision. There are approximately 120 million rod cells and 6 million cone cells, with the cone cells primarily clustered in the central area known as the macula [23]. To maximize photosensitivity, two opposing processes, disc morphogenesis and shedding, renew the numerous stacked membranous discs that make up these organelles on a daily basis [23–25].

Recently, independent studies confirmed that disc morphogenesis originate through evaginations of the ciliary plasma membrane (PM) at the proximal end of the photoreceptor cell [26–28]. In rod photoreceptors, most discs eventually seal off completely to form internalized discrete compartments, while all cone OS discs are continuous with the PM [24]. Photoreceptor cells offset the elongation of their outer segments resulting from continuously adding newly formed discs by actively removing the distal tips of these outer segments. This process, historically referred to as ‘disc shedding’ [29], requires engulfment of the OS distal tip by the underlying retinal pigment epithelium cell (RPE) via receptor-mediated phagocytosis. Furthermore, OS shedding is thought to have evolved to remove oxidative lipid species formed in the light-induced environment in the retina. Given that both the photoreceptors and the RPE are post-mitotic, the synchronized renewal of outer segments of photoreceptors is a fundamental retinal mechanism that maintains perpetual retinal homeostasis and function. Moreover, processing phagosomes in tens of thousands of phagocytic events over the lifetime of a human renders the RPE the most active phagocyte in the body [30].

Considering that cholesterol is a major lipid and ubiquitous constituent of the PM in mammalian cells, the daily renewal of OS discs requires a prodigious supply of cholesterol to reconstruct both the plasma membranes of the RPE and PR. Specifically, cholesterol plays a crucial role in forming and maintaining lipid rafts, which are specialized micro-domains within the plasma membrane (PM). These dynamic microdomains, rich in unesterified cholesterol and glycosphingolipids, function as a specialized surface for cellular receptors to effectively partition and transport proteins and lipids to the cell membrane through a multitude of cellular processes, including cell signalling, endocytosis and exocytosis [21,31,32]. For these and the many other functions relying on cholesterol (see [33]), cells must tightly regulate both the total amount of cellular cholesterol and how it is distributed within and among its membranes.

Mammalian cells have evolved complex regulatory mechanisms to manage cholesterol levels under conditions of cholesterol depletions as well as cholesterol abundance [34]. Cholesterol regulation predominantly occurs in the endoplasmic reticulum (ER), the mitochondria and the plasma membrane (PM), where the coordination of endogenous production, exogenous uptake, removal, storage and chemical modifications supports the maintenance of cellular cholesterol homeostasis [21,35,36].

It is well known that the cholesterol regulating machinery resides in the ER, where key proteins sensing high cellular cholesterol levels respond by inhibiting transcription of cholesterol-promoting pathways. Here, the ER-bound protein SCAP acts as a cholesterol sensor, capturing sterol regulatory element-binding proteins (SREBPs) in the ER when levels are high. Sequestering these transcription factors prevents the activation of genes for cholesterol production and uptake and leads to restored homeostasis, albeit at a slow rate of several hours. Elevated cholesterol levels furthermore evoke post-translational responses mediated by oxysterols, prompting activation of reverse cholesterol transport pathways and subsequent restoration of lipid equilibrium [34,37]. Specifically, 25-hydroxycholesterol (25HC), an oxysterol produced via enzymatic conversion of ER cholesterol, is detected by the membrane domain of HMG-CoA reductase, initiating its binding to the Insig protein. While transcriptional downregulation of cholesterol production occurs relatively slowly, this binding response facilitates the ubiquitylation and degradation of the enzyme to inhibit cholesterol biosynthesis within minutes [38–40].

In addition, oxysterols such as 22(R) -hydroxy cholesterol, 24(S) -hydroxycholesterol and 24(S),25 -epoxycholesterol reduce the uptake of exogenous cholesterol and increase cholesterol efflux by activating the liver X receptor (LXR) through direct binding [41–44]. This subfamily of transcription factors encompasses two isoforms: LXR β , expressed ubiquitously, and LXR α , with expression concentrated in tissues actively involved in lipogenesis. Unlike most Type I nuclear receptors, LXR exhibits constitutive nuclear localization, even in the ligand-free state [21,45]. Ligand-activated LXR, in turn, upregulates the expression of ATP-binding cassette transporter A1 (ABCA1) genes for transcription of the transmembrane protein ABCA1 [34]. ABCA1’s primary role is to facilitate the efflux of intracellular free cholesterol and phospholipids across the plasma membrane to combine with circulating lipid-free apolipoprotein A-I (ApoA-I) and eventually form nascent high-density lipoprotein cholesterol (HDL-C) particles. This process is the initial step in reverse cholesterol transport (RCT), where excess cholesterol from peripheral tissues is removed and transported to the liver for redistribution or excretion by the gallbladder [46]. Finally, activated LXR also upregulates the inducible degrader of LDLR (Idol) to decrease cholesterol uptake from LDL [34,47]. Collectively, these regulatory processes illustrate the complex interactions between various elements involved in maintaining cholesterol homeostasis.

In our recent work [21], we provide detailed descriptions of cholesterol regulation pathways at the cellular level.

### 2.1. Cholesterol pathways in the RPE

Considering cholesterol sources in the retina, independent studies by Rao *et al*. [48], Biswas *et al*. [49] and Louer *et al*. [50] qualitatively demonstrated *de novo* synthesis of cholesterol in RPE cells. (The interested reader is referred to a summary of key molecules and steps involved in the cholesterol biosynthesis pathway in [51]). At the same time, systemic delivery of cholesterol is achieved via the uptake of low-density lipoproteins (LDL) and high-density lipoproteins (HDL) by LDL receptors (LDLR) and scavenger receptors (SR-BI and SR-BII), respectively, which are located in the RPE plasma membrane [52,53]. While the balance of absolute sterol acquisition rates between these two pathways in the RPE is currently unknown, the key regulator of gene expression to realize these pathways is the ER membrane protein called SCAP [21,54]. Specifically, Louer *et al*. [50] confirmed the expression of the mevalonate pathway gene, HMGCR, in the RPE, where expression of this gene is dependent on the regulated release of the SREBP/SCAP protein complex and its associated transcription factors to the Golgi [37]. Similarly, the transcription factors required for gene expression of LDLR and SR-BII receptors are regulated through the exact SREBP/SCAP mechanism [37,53].

Following the daily phagocytosis of the outer segment disc membrane into a nascent phagosome, the phagosome matures by migrating to the basal side of the RPE while undergoing gradual acidification and degradation through phagolysosomal mechanisms to release unesterified cholesterol (UC) [55]. Evidence exists that this cholesterol, together with any excess UC in the RPE, could be removed via four efflux pathways: (i) apical efflux via ABCA1 transporters into the subretinal space [56,57]; (ii) basal efflux to the circulation [51,57]; (iii) localized enzymatic conversion to oxysterols [58]; and (iv) basolateral release as part of a unique APoB-containing lipoprotein into the pre-BlinD layer, from where it enters the circulation via the choriocapillaris, as described in §1 and shown in figure 2 [9,59].

### 2.2. Cholesterol pathways in the neural retina

A recent review by Rao and Fliesler [33] summarizes strong evidence from their earlier studies [60–62] and from other more recent studies [63–65], that the vertebrate retina can synthesize cholesterol *de novo*. Furthermore, based on their review, multiple cell types of the retina most likely have this capacity, including RPE cells, the PR cells and Müller glia.

Significantly, a most recent study, involving *in vivo* imaging of the mouse retina to track lipoprotein particles and lipid distribution, provides an explanation of why *in situ* biosynthesis is the major source of cholesterol for the retina; their data indicates limited trafficking of serum lipoprotein particles (LPP) from the RPE to the neural retina, thus it seems to stimulate *de novo* synthesis of cholesterol in the neural retina [7].

Investigating the mechanisms underlying cholesterol biosynthesis and regulation of cholesterol through uptake/efflux pathways in Müller cells, data obtained by Léger-Charnay *et al*. [66] indicates regulation of multiple *de novo* cholesterol synthesis pathway genes, including HMGCR and SREBP2 via 24(S)-hydroxycholesterol, suggesting that Müller cells can efficiently synthesize cholesterol endogenously. Furthermore, their data suggests that Müller cells express the molecular machinery, that is, ABCA1 and ApoE genes, to regulate cholesterol efflux through secretion of ApoE and HDL particles to the subretinal space surrounding neurons.

Given that the interphotoreceptor matrix, a highly organized structure with interconnected domains surrounding cone and rod photoreceptor cells, extend throughout the subretinal space, the cholesterol-carrying HDL particles could be taken up via SR-BI receptors on the PR plasma membrane [2,4]. Significantly, SR-BI gene expression is regulated by SREBP-1 transcription factors in response to altered intracellular sterol levels [67]. Immunohistochemical analysis of the sterol homeostasis machinery at the protein level reveals that HMGCR, SREBP and SCAP, the proteins of the mevalonate pathway, are expressed in PRs [4]. Furthermore, transcript expression profiles of genes encoding the fundamental mevalonate pathway enzymes during retinal development confirms the capacity of PRs to synthesis cholesterol *de novo* [4,33].

Abbreviations used throughout this work are presented in table 1.

**Table 1.** List of abbreviations.

list of abbreviations	
ABCA1	ATP-binding cassette subfamily A member 1
ACAT	acyl-coenzyme A (CoA):cholesterol acyltransferase
CE	esterified cholesterol
COPII	coat protein complex II
DHCR7	7-dehydrocholesterol reductase
DHCR24	24-dehydrocholesterol reductase
ER	endoplasmic reticulum
HDL	high-density lipoprotein
HMG-CoA	3-hydroxy-3-methylglutaryl-coenzyme A
HMGCR	enzyme 3-hydroxy-3-methylglutaryl-coenzyme A reductase
Idol	inducible degrader of LDLR
LDL	low-density lipoprotein
LDLR	low-density lipoprotein receptor
MVK	mevalonate kinase
PCSK9	proprotein convertase subtilisin/kexin type-9
PFO	perfringolysin O
PM	plasma membrane
SCAP	SREBP-cleavage activating protein
SCC	strongly connected component
SREBP	sterol regulatory element-binding proteins
SR–BI	scavenger receptor, class B type 1
TF	transcription factor
25HC	25-hydroxycholesterol
27HC	27-hydroxycholesterol
MVK	mevalonate kinase
MG	Müller glia

## 3. Methods

### 3.1. A chemical reaction network of retinal cholesterol homeostasis

In earlier studies [21], we established a comprehensive framework for investigating the necessary biochemical conditions for robust homeostasis and adaptation in complex biological networks, such as the tight regulation of cholesterol that is required in the retinal layers of the human eye. In §2, we confirmed that gene transcription for the proteins involved in regulating cholesterol concentration via the antithetic integral control mechanism, as originally identified in [21], also exist in the cellular layers of the retina. Here we provide a description of how these intricate biochemical processes can be represented as a collection of chemical reactions to generate an overarching chemical reaction network (CRN) for the regulation of cholesterol at the intracellular level and intercellular level in the human retina.

The nomenclature used to represent the species in these reactions is an extended form of the original representation used in Scheepers and Araujo [21], to allow continuity in concept and also to define new molecular species in a similar form. As such, for the generalized species $S_{i}$ or $S^{i}$ , species in the RPE are denoted with i=0 , the species in the PRs with i=1 and the species in the MG with i=2 . A list of 45 identified molecular species and their associated symbols are provided in table 2 (species unique to the retinal cholesterol regulatory network are indicated in blue text).

**Table 2.** Species in the RPE, PR and MG.

species	RPE	PR	MG
SREBP/Scap/Insig complex	$S_{c\,i}^{0}$	$S_{c\,i}^{1}$	$S_{c\,i}^{2}$
active cholesterol	$C_{0}$	$C_{1}$	$C_{2}$
cholesterol in ER membrane	$C_{e}^{0}$	$C_{e}^{1}$	$C_{e}^{2}$
cholesterol in PM	$C_{p}^{0}$	$C_{p}^{1}$	$C_{p}^{2}$
cholesterol-carrying LDL	$C_{L}^{0}$		$C_{L}^{2}$
cholesterol-carrying HDL from circulation	$C_{H}^{0}$		
cholesterol-carrying HDL from interphotoreceptor matrix to PR		$C_{H}^{1}$	
LDL receptor (LDLR)	$R_{0}$		$R_{2}$
HDL receptor (SR-BI)	$R_{b}^{0}$	$R_{b}^{1}$	
cholesterol released from receptors	$C_{f}^{0}$	$C_{f}^{1}$	$C_{f}^{2}$
esterified cholesterol	$E_{0}$	$E_{1}$	
3-hydroxy-3-methylglutaryl-coenzyme A (HMG-CoA)	$H_{0}$	$H_{1}$	$H_{2}$
3-hydroxy-3-methylglutaryl-coenzyme A reductase (HMGCR)	$H_{R}^{0}$	$H_{R}^{1}$	$H_{R}^{2}$
proprotein convertase subtilisin/kexin type-9 (PCSK9)	$P_{0}$		$P_{2}$
SREBP transcription factor for LDLR	$S_{r}^{0}$		$S_{r}^{2}$
SREBP transcription factor for SR-B1	$S_{b}^{0}$	$S_{b}^{1}$	
SREBP transcription factor for HMGCR	$S_{h}^{0}$	$S_{h}^{1}$	$S_{h}^{2}$
SREBP transcription factor for PCSK9	$S_{p}^{0}$		$S_{p}^{2}$
cholesterol in outer segments of PR		$C_{O}$	
cholesterol in the phagosome	$C_{g}$		
ApoB-lipoprotein	B		

#### 3.1.1. Biochemical reactions in the Müller glia (MG)

The molecular interactions associated with cholesterol regulation in the MG correspond to those identified in our earlier work [21], where the endogenous production and exogenous uptake pathways of cholesterol are tightly regulated via the sensor molecule, $C_{e}^{2}$ , to either sequestrate the SREBP/SCAP complex ( $S_{ci}^{2}$ ) or, under depleted cholesterol conditions, facilitate SREBP transcription factor release to the Golgi. Similar to our previous work, we model this irreversible inhibition reaction as $S_{ci}^{2}+C_{2}\overset{\epsilon }{\to }\emptyset$ . The chemical reactions for the release of the relevant transcription factors and subsequent gene expression for LDLR ( $R_{2}$ ), PSCK9 ( $P_{2}$ ) and HMGCR ( $H_{R}^{2}$ ) again follow the same modelling regime as our prior work, albeit with adjusted symbols to represent the process in the MG. Exogenous cholesterol input to the MG, $C_{L}^{2}$ , occurs via LDLR, which in turn is tightly regulated by the $P_{2}$ inhibitor enzyme. The cholesterol released by endolysosomes, $C_{f}^{2}$ , intercalates with phospholipids to form part of the PM cholesterol, $C_{p}^{2}$ , while it is postulated that cholesterol fluxes between PM and ER maintain cholesterol pool concentration in both cellular levels [68]. The endogenous production of cholesterol is modelled as the conversion of HMG-CoA, $H_{2}$ , to ER cholesterol, $C_{e}^{2}$ , under tight control of the rate-limiting enzyme $H_{R}^{2}$ . The enzyme $H_{R}^{2}$ , in turn, is regulated via the presence of oxysterol to facilitate ubiquitylation and degradation of the enzyme under abundant cholesterol conditions. This cholesterol-initiated degradation process is modelled as $C_{e}^{2}+H_{R}^{2}\overset{k_{15}}{\to }C_{e}^{2}$ in the MG, and represented in a similar way in the PRs and RPE. (Note that we capture the presence of oxysterols implicitly via its source, ER cholesterol ( $C_{e}$ ).)

Excess cholesterol effluxes via HDL particles into the sub-retinal space is modelled here as $C_{e}^{2}\overset{p_{13}}{\to }\emptyset$ , where it is available for uptake by the photoreceptors, if required. Full chemical reaction kinetics for all biochemical reactions in the MG are summarized in column three of table 3.

**Table 3.** Biochemical reactions of the retinal cholesterol regulation network.

molecular processes modelled	RPE	PR	NR
constitutive production of SREBP/Scap/Insig in ER	$\emptyset \overset{𝜇}{\to }S_{c\,i}^{0}$	$\emptyset \overset{\phi }{\to }S_{c\,i}^{1}$	$\emptyset \overset{𝛽}{\to }S_{c\,i}^{2}$
proportion of cholesterol released into ER-lumen	$C_{e}^{0}\overset{𝜃}{\to }C_{e}^{0}+C_{0}$	$C_{e}^{1}\overset{𝜆}{\to }C_{e}^{1}+C_{1}$	$C_{e}^{2}\overset{𝜅}{\to }C_{e}^{2}+C_{2}$
SREBP/Scap/Insig/cholesterol retention in ER	$S_{c\,i}^{0}+C_{0}\overset{𝜂}{\to }\emptyset$	$S_{c\,i}^{1}+C_{1}\overset{𝜓}{\to }\emptyset$	$S_{c\,i}^{2}+C_{2}\overset{\epsilon }{\to }\emptyset$
SREBP/Scap complex releases transcription factors in Golgi	$S_{ci}^{0}\overset{k_{1}}{\to }S_{ci}^{0}+S_{r}^{0}+S_{h}^{0}+S_{p}^{0}+S_{b}^{0}$	$S_{ci}^{1}\overset{p_{1}}{\to }S_{ci}^{1}+S_{b}^{1}+S_{h}^{1}$	$S_{ci}^{2}\overset{p_{26}}{\to }S_{ci}^{2}+S_{h}^{2}+S_{r}^{2}+S_{p}^{2}$
gene expression for LDLR	$S_{r}^{0}\overset{k_{17}}{\to }S_{r}^{0}+R_{0}$		$S_{r}^{2}\overset{p_{33}}{\to }S_{r}^{2}+R_{2}$
gene expression for HMGCR	$S_{h}^{0}\overset{k_{18}}{\to }S_{h}^{0}+H_{R}^{0}$	$S_{h}^{1}\overset{p_{7}}{\to }S_{h}^{1}+H_{R}^{1}$	$S_{h}^{2}\overset{p_{19}}{\to }S_{h}^{2}+H_{R}^{2}$
gene expression for PCSK9	$S_{p}^{0}\overset{k_{19}}{\to }S_{p}^{0}+P_{0}$		$S_{p}^{2}\overset{p_{37}}{\to }S_{p}^{2}+P_{2}$
gene expression for SR-BI receptor	$S_{b}^{0}\overset{k_{28}}{\to }S_{b}^{0}+R_{b}^{0}$	$S_{b}^{1}\overset{p_{9}}{\to }S_{b}^{1}+R_{b}^{1}$	
LDLR transcription factor degradation	$S_{r}^{0}\overset{k_{2}}{\to }\emptyset$		$S_{r}^{2}\overset{p_{32}}{\to }\emptyset$
HMGCR transcription factor degradation	$S_{h}^{0}\overset{k_{10}}{\to }\emptyset$	$S_{h}^{1}\overset{p_{3}}{\to }\emptyset$	$S_{h}^{2}\overset{p_{18}}{\to }\emptyset$
PCSK9 transcription factor degradation	$S_{p}^{0}\overset{k_{13}}{\to }\emptyset$	$S_{p}^{2}\overset{p_{31}}{\to }\emptyset$	
SR-BI transcription factor degradation	$S_{b}^{0}\overset{k_{27}}{\to }\emptyset$	$S_{b}^{1}\overset{p_{2}}{\to }\emptyset$	
receptor-mediated uptake and release of free LDL cholesterol	$R_{0}+C_{L}^{0}\overset{k_{3}}{\to }R_{0}+C_{L}^{0}+C_{f}^{0}$		$R_{2}+C_{L}^{2}\overset{p_{34}}{\to }R_{2}+C_{L}^{2}+C_{f}^{2}$
receptor-mediated uptake and release of free HDL cholesterol	$R_{b}^{0}+C_{H}^{0}\overset{k_{26}}{\to }R_{b}^{0}+C_{H}^{0}+C_{f}^{0}$	$R_{b}^{1}+C_{H}^{1}\overset{p_{25}}{\to }R_{b}^{1}+C_{H}^{1}+C_{f}^{1}$	
Idol-mediated uibiquitylation and degradation of LDLR	$R_{0}\overset{k_{11}}{\to }\emptyset$		$R_{2}\overset{p_{35}}{\to }\emptyset$
degradation of SR-BI receptor	$R_{b}^{0}\overset{k_{29}}{\to }\emptyset$	$R_{b}^{1}\overset{p_{10}}{\to }\emptyset$	
PCSK9-mediated degradation of LDLR	$P_{0}+R_{0}\overset{k_{14}}{\to }P_{0}\overset{k_{16}}{\to }\emptyset$		$P_{2}+R_{2}\overset{p_{39}}{\to }P_{2}\overset{p_{38}}{\to }\emptyset$
free cholesterol transferred to PM cholesterol	$C_{f}^{0}\overset{k_{4}}{\to }C_{p}^{0}$	$C_{f}^{1}\overset{p_{4}}{\to }C_{p}^{1}$	$C_{f}^{2}\overset{p_{36}}{\to }C_{p}^{2}$
PR PM evagination to form PR OS membrane		$C_{p}^{1}\overset{p_{11}}{\to }C_{O}$	
cholesterol distribution between PM and ER	$C_{p}^{0}\overset{k_{5}}{\underset{k_{6}}{⇌}}C_{e}^{0}$	$C_{p}^{1}\overset{p_{5}}{\underset{p_{6}}{⇌}}C_{e}^{1}$	$C_{p}^{2}\overset{p_{28}}{\underset{p_{29}}{⇌}}C_{e}^{2}$
cholesterol esterification & hydrolysis	$C_{e}^{0}\overset{k_{7}}{\underset{k_{8}}{⇌}}E_{0}$	$C_{e}^{1}\overset{p_{16}}{\underset{p_{17}}{⇌}}E_{1}$	
constitutive production of HMGCoA	$\emptyset \overset{\alpha }{\to }H_{0}$	$\emptyset \overset{\omega }{\to }H_{1}$	$\emptyset \overset{\delta }{\to }H_{2}$
endogenous production of cholesterol	$H_{0}+H_{R}^{0}\overset{k_{9}}{\to }H_{R}^{0}+C_{e}^{0}$	$H_{1}+H_{R}^{1}\overset{p_{12}}{\to }H_{R}^{1}+C_{e}^{1}$	$H_{2}+H_{R}^{2}\overset{p_{21}}{\to }H_{R}^{2}+C_{e}^{2}$
ubiquitylation and degradation of sterol bound HMGCR	$C_{e}^{0}+H_{R}^{0}\overset{k_{15}}{\to }C_{e}^{0}$	$C_{e}^{1}+H_{R}^{1}\overset{p_{8}}{\to }C_{e}^{1}$	$C_{e}^{2}+H_{R}^{2}\overset{p_{20}}{\to }C_{e}^{2}$
phagocytosis of PR-OS tips	$C_{p}^{0}+C_{O}\overset{k_{12}}{\to }C_{g}$		
cholesterol efflux via ABCA1 transporters into HDL particles	$C_{e}^{0}\overset{k_{21}}{\to }\emptyset$	$C_{e}^{1}\overset{p_{15}}{\to }\emptyset$	$C_{e}^{2}\overset{p_{13}}{\to }\emptyset$
cholesterol removal via ApoB-lipoprotein	$C_{e}^{0}+C_{g}\overset{k_{22}}{\to }B\overset{k_{24}}{\to }\emptyset$		

#### 3.1.2. Biochemical reactions in the photoreceptors

Considering the recent hypothesis by El-Darzi *et al*. [7] that the neural retina synthesizes all cholesterol required endogenously instead of receiving some cholesterol from the circulation via the RPE, we again model the biochemical signalling pathways of cholesterol regulation in the PRs based on the model designed in [21], with the exception that cholesterol input into the PR cell is via the HDL particle, $C_{H}^{1}$ , carrying cholesterol released from the MG. Uptake of the HDL particle is facilitated by the SR-BI receptor, $R_{b}^{1}$ , on the cell membrane of the PR, and modelled here as $R_{b}^{1}+C_{H}^{1}\overset{p_{25}}{\to }R_{b}^{1}+C_{H}^{1}+C_{f}^{1}$ . Remarkably, evidence exists that the transcription factor for gene activation of the SR-BI protein is part of the SREBP family of transcription factors [53] and, as such, also dependent on the release of SREBP transcription factors ( $S_{ci}^{1}$ ) in the PR under cholesterol depleted conditions, as postulated previously. In addition, the consistent transfer of cholesterol from the PR plasma membrane evaginations to form the outer segment discs of the PRs, are modelled as $C_{p}^{1}\overset{p_{11}}{\to }C_{O}$ . Full chemical reaction kinetics for all the biochemical reactions identified in the PR are summarized in column two of table 3.

#### 3.1.3. Biochemical reactions in the RPE

As identified in the literature and described in §2, the full set of molecular species identified to regulate cholesterol homeostasis robustly in the mammalian cell also exists in the RPE cell layer. In addition, it has been shown that the RPE also take up cholesterol-carrying HDL particles from the choriocappilaris via SR-BI receptors ( $R_{b}$ ), with similar transcription and gene activation pathways as previously described in §3.1.2. We model this uptake of cholesterol as $R_{b}^{0}+C_{H}^{0}\overset{k_{26}}{\to }R_{b}^{0}+C_{H}^{0}+C_{f}^{0}$ . In addition, the daily phagocytosis of PR OS membrane tips involves engulfment from the RPE PM, and the reaction equation representing this process is $C_{p}^{0}+C_{O}\overset{k_{12}}{\to }C_{g}$ , where $C_{g}$ represents the cholesterol in the phagosome in the RPE. The cholesterol released from the phagosome, together with any excess ER cholesterol, are removed to the choriocapillaris basolaterally and this process is modelled as $C_{e}^{0}+C_{g}\overset{k_{22}}{\to }B\overset{k_{22}}{\to }\emptyset$ .

**Figure 3.**
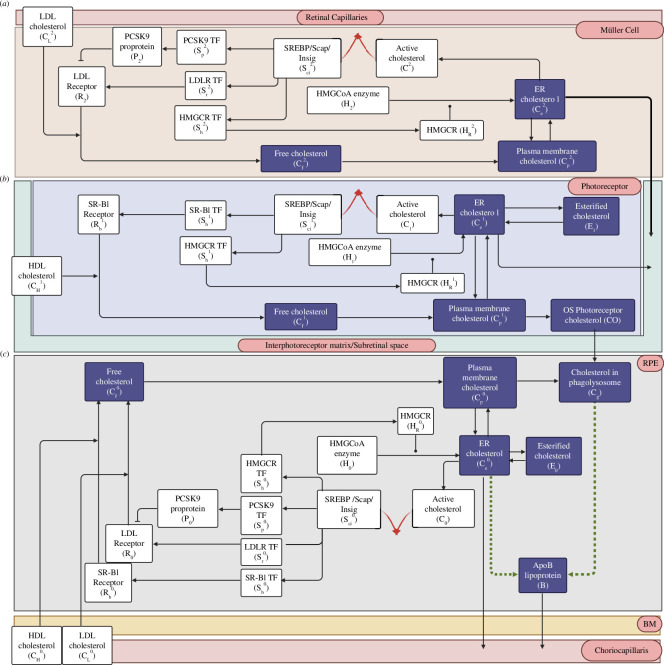
Network schematic for cellular cholesterol regulation in the retina. The presented network diagram captures the interplay between the 41 entities of the CRN, unveiling the fundamental organizational structure of the cellular cholesterol homeostatic apparatus as a singular Opposer module in (*a*), the MG, (*b*), the PRs and (*c*), the RPE, characterized by its distinctive feedback architecture. Arrows depict flux (activation), blunt ends depict inhibition and solid circles denote catalysis. Red arrows signify the opposing nodes: irreversible cholesterol sequestration in the $S_{ci}^{0}C_{0}$ , $S_{ci}^{1}C_{1}$ and $S_{ci}^{2}C_{2}$ complexes. Dashed dark green arrows represent the proposed assembly of a unique ApoB lipoprotein particle characteristic to the retina. (Created with BioRender.com.)

Together, 45 molecular species across the MG, PRs, RPE, BM and CC participate in 41 biochemical reactions to collectively generate the retinal cholesterol CRN. Table 2 provides a list of the 45 species and their associated symbols used in the reactions while the full collection of biochemical reactions is listed in table 3. Figure 3 presents a schematic summary of these interactions, outlining the overall network organization for regulating the concentration of cholesterol in the retina.

### 3.2. Chemical reaction rates

The network of chemical reactions established in §3.1 induces the following polynomial dynamical system of 41 reaction rates, which together capture the dynamical regulation of cholesterol concentration in the RPE and neural layers of the retina:


(3.1) $$\begin{array}{rl} h_{1} & =\frac{dS_{ci}^{0}}{dt}=\mu -\eta S_{ci}^{0}C^{0}\;, \end{array}$$

(3.2) $$\begin{array}{rl} h_{2} & =\frac{dC_{0}}{dt}=\theta C_{e}^{0}-\eta S_{ci}^{0}C_{0}\;, \end{array}$$

(3.3) $$\begin{array}{rl} h_{3} & =\frac{dS_{r}^{0}}{dt}=k_{1}S_{ci}^{0}-k_{2}S_{r}^{0}\;, \end{array}$$

(3.4) $$\begin{array}{rl} h_{4} & =\frac{dS_{h}^{0}}{dt}=k_{1}S_{ci}^{0}-k_{10}S_{h}^{0}\;, \end{array}$$

(3.5) $$\begin{array}{rl} h_{5} & =\frac{dR_{0}}{dt}=k_{17}S_{r}^{0}-k_{11}R_{0}-k_{14}P_{0}R_{0}\;, \end{array}$$

(3.6) $$\begin{array}{rl} h_{6} & =\frac{dC_{f}^{0}}{dt}=k_{3}C_{L}^{0}R_{0}+k_{26}C_{H}^{0}R_{b}^{0}-k_{4}C_{f}^{0}\;, \end{array}$$

(3.7) $$\begin{array}{rl} h_{7} & =\frac{dC_{p}^{0}}{dt}=k_{4}C_{f}^{0}-k_{5}C_{p}^{0}+k_{6}C_{e}^{0}-k_{12}C_{p}^{0}C_{O}\;, \end{array}$$

(3.8) $$\begin{array}{rl} h_{8} & =\frac{dC_{e}^{0}}{dt}=k_{5}C_{p}^{0}-k_{6}C_{e}^{0}+k_{9}H_{R}^{0}H_{0}-k_{7}C_{e}^{0}+k_{8}E_{0}-k_{21}C_{e}^{0}-k_{22}C_{e}^{0}C_{g}\;, \end{array}$$

(3.9) $$\begin{array}{rl} h_{9} & =\frac{dE_{0}}{dt}=k_{7}C_{e}^{0}-k_{8}E_{0}\;, \end{array}$$

(3.10) $$\begin{array}{rl} h_{10} & =\frac{dH_{R}^{0}}{dt}=k_{18}S_{h}^{0}-k_{15}H_{R}^{0}C_{e}^{0}\;, \end{array}$$

(3.11) $$\begin{array}{rl} h_{11} & =\frac{dH_{0}}{dt}=\alpha -k_{9}H_{R}^{0}H_{0}\;, \end{array}$$

(3.12) $$\begin{array}{rl} h_{12} & =\frac{dS_{p}^{0}}{dt}=k_{1}S_{ci}^{0}-k_{13}S_{p}^{0}\;, \end{array}$$

(3.13) $$\begin{array}{rl} h_{13} & =\frac{dP_{0}}{dt}=k_{19}S_{p}^{0}-k_{16}P_{0}\;, \end{array}$$

(3.14) $$\begin{array}{rl} h_{14} & =\frac{dS_{b}^{0}}{dt}=k_{1}S_{ci}^{0}-k_{27}S_{b}^{0}\;, \end{array}$$

(3.15) $$\begin{array}{rl} h_{15} & =\frac{dR_{b}^{0}}{dt}=k_{28}S_{b}^{0}-k_{29}R_{b}^{0}\;, \end{array}$$

(3.16) $$\begin{array}{rl} h_{16} & =\frac{dC_{f}^{1}}{dt}=p_{25}C_{H}^{1}R_{b}^{1}-p_{4}C_{f}^{1}\;, \end{array}$$

(3.17) $$\begin{array}{rl} h_{17} & =\frac{dS_{ci}^{1}}{dt}=\phi -\psi S_{ci}^{1}C_{1}\;, \end{array}$$

(3.18) $$\begin{array}{rl} h_{18} & =\frac{dC_{1}}{dt}=\lambda C_{e}^{1}-\psi S_{ci}^{1}C_{1}\;, \end{array}$$

(3.19) $$\begin{array}{rl} h_{19} & =\frac{dS_{b}^{1}}{dt}=p_{1}S_{ci}^{1}-p_{2}S_{b}^{1}\;, \end{array}$$

(3.20) $$\begin{array}{rl} h_{20} & =\frac{dS_{h}^{1}}{dt}=p_{1}S_{ci}^{1}-p_{3}S_{h}^{1}\;, \end{array}$$

(3.21) $$\begin{array}{rl} h_{21} & =\frac{dR_{b}^{1}}{dt}=p_{9}S_{b}^{1}-p_{10}R_{b}^{1}\;, \end{array}$$

(3.22) $$\begin{array}{rl} h_{22} & =\frac{dH_{R}^{1}}{dt}=p_{7}S_{h}^{1}-p_{8}H_{R}^{1}Ce^{1}\;, \end{array}$$

(3.23) $$\begin{array}{rl} h_{23} & =\frac{dC_{p}^{1}}{dt}=p_{4}C_{f}^{1}-p_{5}C_{p}^{1}+p_{6}C_{e}^{1}-p_{11}C_{p}^{1}\;, \end{array}$$

(3.24) $$\begin{array}{rl} h_{24} & =\frac{dC_{e}^{1}}{dt}=p_{5}C_{p}^{1}-p_{6}C_{e}^{1}-p_{16}C_{e}^{1}+p_{17}E_{1}+p_{12}H_{R}^{1}H_{1}-p_{15}Ce^{1}\;, \end{array}$$

(3.25) $$\begin{array}{rl} h_{25} & =\frac{dE_{1}}{dt}=p_{16}C_{e}^{1}-p_{17}E_{1}\;, \end{array}$$

(3.26) $$\begin{array}{rl} h_{26} & =\frac{dH_{1}}{dt}=\omega -p_{12}H_{R}^{1}H_{1}\;, \end{array}$$

(3.27) $$\begin{array}{rl} h_{27} & =\frac{dC_{O}}{dt}=p_{11}C_{p}^{1}-k_{12}C_{p}^{0}C_{O}\;, \end{array}$$

(3.28) $$\begin{array}{rl} h_{28} & =\frac{dC_{g}}{dt}=k_{12}C_{p}^{0}C_{O}-k_{22}C_{e}^{0}C_{g}\;, \end{array}$$

(3.29) $$\begin{array}{rl} h_{29} & =\frac{dB}{dt}=k_{22}C_{e}^{0}C_{g}-k_{24}B\;, \end{array}$$

(3.30) $$\begin{array}{rl} h_{30} & =\frac{dS_{ci}^{2}}{dt}=\beta -\epsilon S_{ci}^{2}C_{2}\;, \end{array}$$

(3.31) $$\begin{array}{rl} h_{31} & =\frac{dC_{2}}{dt}=\kappa C_{e}^{2}-\epsilon S_{ci}^{2}C_{2}\;, \end{array}$$

(3.32) $$\begin{array}{rl} h_{32} & =\frac{dS_{r}^{2}}{dt}=p_{26}S_{ci}^{2}-p_{32}S_{r}^{2}\;, \end{array}$$

(3.33) $$\begin{array}{rl} h_{33} & =\frac{dS_{h}^{2}}{dt}=p_{26}S_{ci}^{2}-p_{18}S_{h}^{2}\;, \end{array}$$

(3.34) $$\begin{array}{rl} h_{34} & =\frac{dS_{p}^{2}}{dt}=p_{26}S_{ci}^{2}-p_{31}S_{p}^{2}\;, \end{array}$$

(3.35) $$\begin{array}{rl} h_{35} & =\frac{dR_{2}}{dt}=p_{33}S_{r}^{2}-p_{35}R_{2}-p_{39}P_{2}R_{2}\;, \end{array}$$

(3.36) $$\begin{array}{rl} h_{36} & =\frac{dP_{2}}{dt}=p_{37}S_{p}^{2}-p_{38}P_{2}\;, \end{array}$$

(3.37) $$\begin{array}{rl} h_{37} & =\frac{dC_{f}^{2}}{dt}=p_{34}C_{L}^{2}R_{2}-p_{36}C_{f}^{2}\;, \end{array}$$

(3.38) $$\begin{array}{rl} h_{38} & =\frac{dC_{p}^{2}}{dt}=p_{36}C_{f}^{2}-p_{28}C_{p}^{2}+p_{29}Ce^{2}\;, \end{array}$$

(3.39) $$\begin{array}{rl} h_{39} & =\frac{dC_{e}^{2}}{dt}=p_{28}C_{p}^{2}-p_{29}C_{e}^{2}+p_{21}H_{R}^{2}H_{2}-p_{13}C_{e}^{2}\;, \end{array}$$

(3.40) $$\begin{array}{rl} h_{40} & =\frac{dH_{2}}{dt}=\delta -p_{21}H_{R}^{2}H_{2}\;, \end{array}$$

(3.41) $$\begin{array}{rl} h_{41} & =\frac{dH_{R}^{2}}{dt}=p_{19}S_{h}^{2}-p_{20}H_{R}^{2}C_{e}^{2}\;.\; \end{array}$$

Recent theoretical advances on the general principles governing RPA in arbitrarily large and complex CRNs throughout biology [18,19,69–71] now allow us to implement a systematic analysis of RPA capacity of cholesterol concentrations in the highly complex multi-compartment CRN governing cholesterol homeostasis in the retina. In particular, the Kinetic Pairing Theorem (see Theorem 1 in [19]) establishes that for every RPA-capable CRN, with interacting molecules $x_{1},...,x_{n}$ and corresponding reaction rates $r_{1},...,r_{n}$ , it is possible to find a set of polynomials $\left\{h_{1},...,h_{n}\}\subset \mathbb{R}[x_{1},...,x_{n}\right]$ such that


(3.42) $$\begin{array}{ccc} & h_{1}r_{1}+...+h_{n}r_{n}=f(x_{i},x_{j})(x_{i}-k)=\sigma , & \end{array}$$

where $x_{i}$ is any RPA-capable molecule in the CRN, with setpoint k , and $x_{j}$ is any non-RPA-capable molecule in the CRN. In its simplest form, $\sigma =f(x_{i},x_{j})(x_{i}-k)$ constitutes the RPA polynomial of the CRN, a distinguished polynomial in two (and only two) variables, with $f(x_{i},x_{j})$ its associated kinetic pairing function. Importantly, for reasons related to the underlying linearity of the CRN’s graph structure, the RPA polynomial, if it exists for the CRN under consideration, is always decomposable into a topological hierarchy of linear invariants, each obtained through only ℝ -linear combinations of the system’s rate equations. This guarantees that the Gröbner basis computation required to test the existence of an RPA polynomial may be executed in polynomial time, comparable with Gaussian elimination. Importantly, this algebro-geometric algorithm allows for the analysis of RPA capacity while treating parameters symbolically, enabling us to draw important conclusions on the qualitative responses of the network to various possible perturbations without knowing numerical values for any parameters in advance.

## 4. Results

### 4.1. Polynomial analysis of the retinal cholesterol CRN

Using the methodology described in §3.2, we systematically test each molecular species for RPA, using the input molecules ( $C_{L}^{0}$ , $C_{H}^{0}$ , $C_{H}^{1}$ , $C_{L}^{2}$ ) as the non-RPA-capable species in each case of the application of the Kinetic Pairing Theorem [19]. We have developed an open-source code for the implementation of this calculation, in the freely available software Singular (https://www.singular.uni-kl.de/) within the interactive Jupyter environment. Data and relevant code for this research work are stored in GitHub: https://github.com/RonelScheepers/RPAinRetina.git and have been archived within the Zenodo repository: https://zenodo.org/doi/10.5281/zenodo.13334168. We also refer interested readers to our detailed discussion of the implementation of this systematic method for RPA-detection [21] in the context of the much simpler case of single-cell-level cholesterol regulation.

This systematic method allows us to partition the species of the CRN into two disjoint sets of RPA-capable species, and non-RPA-capable species. We give a full listing of the RPA-capable species in table 4. Our Singular code also automatically computes the setpoint of each RPA-capable species (also given in table 4), as well as the combination of polynomials in the set $h_{1},....,h_{n}$ (see equation (3.42)) required to project the reaction rates onto the RPA polynomial. For instance, the linear combination of rate equations that identify the RPA polynomial within the steady state ideal for the ApoB-lipoprotein particle ( B ), the particle suggested by Curcio *et al*. [9] to be the key contributor of cholesterol in drusen, is revealed by the lift function in Singular to be:


(4.1) $$\begin{array}{ccc} & \begin{array}{cc} & (p_{6}p_{11}+p_{11}p_{15})\frac{dC_{1}}{dt}-(p_{6}p_{11}+p_{11}p_{15})\frac{dS_{ci}^{1}}{dt}+p_{11}\lambda \frac{dC_{e}^{1}}{dt}+p_{11}\lambda \frac{E_{1}}{dt}+p_{11}\lambda \frac{dH_{1}}{dt}\\ & -p_{5}\lambda \frac{dC_{O}}{dt}-p_{5}\lambda \frac{dC_{g}}{dt}-p_{5}\lambda \frac{dB}{dt}\;. \end{array} & \end{array}$$

**Table 4.** RPA-capable species and their setpoints.

$C_{e}^{0}=\frac{\mu }{\theta }$
$C_{p}^{0}=\frac{k_{6}p_{5}\mu \lambda +k_{21}p_{5}\mu \lambda -p_{5}\theta \alpha \lambda +p_{6}p_{11}\theta \phi +p_{11}p_{15}\theta \phi -p_{11}\theta \lambda \omega }{k_{5}p_{5}\theta \lambda }$
$E_{0}=\frac{k_{7}\mu }{k_{8}\theta }$
$C_{f}^{0}=\frac{k_{21}p_{5}\mu \lambda -p_{5}\theta \alpha \lambda +2(p_{6})p_{11}\theta \phi +2(p_{11})p_{15}\theta \phi +2(p_{11})\theta \lambda \omega }{k_{4}p_{5}\theta \lambda }$
$C_{g}=\frac{p_{6}p_{11}\theta \phi +p_{11}p_{15}\theta \phi -p_{11}\theta \lambda \omega }{k_{22}p_{5}\mu \lambda }$
$B=\frac{p_{6}p_{11}\phi +p_{11}p_{15}\phi -p_{11}\lambda \omega }{k_{24}p_{5}\lambda }$
$C_{e}^{1}=\frac{\phi }{\lambda }$
$C_{p}^{1}=\frac{p_{6}\phi +p_{15}\phi -\lambda \omega }{p_{5}\lambda }$
$C_{f}^{1}=\frac{p_{5}p_{15}\phi -p_{5}\lambda \omega +p_{6}p_{1}\phi +p_{11}p_{15}\phi -p_{11}\lambda \omega }{p_{4}p_{5}\lambda }$
$E_{1}=\frac{p_{16}\phi }{p_{17}\lambda }$
$C_{O}=\frac{k_{5}p_{6}p_{11}\theta \phi +k_{5}p_{11}p_{15}\theta \phi -k_{5}p_{11}\theta \lambda \omega }{k_{6}k_{12}p_{5}\mu \lambda +k_{12}k_{21}p_{5}\mu \lambda -k_{12}p_{5}\theta \alpha \lambda +k_{12}p_{6}p_{11}\theta \phi +k_{12}p_{11}p_{15}\theta \phi -k_{12}p_{11}\theta \lambda \omega }$
$C_{e}^{2}=\frac{\beta }{\kappa }$
$C_{p}^{2}=\frac{p_{13}\beta +p_{29}\beta -\kappa \delta }{p_{28}\kappa }$
$C_{f}^{2}=\frac{p_{13}\beta -\kappa \delta }{p_{36}\kappa }$

To identify which molecules are directly regulated by an RPA-conferring control mechanism, and which inherit the RPA property indirectly as a result of their topological relationship to the molecules under direct homeostatic control, we have undertaken a detailed graphical analysis of the CRN. To provide context for this graphical approach, we note that recent research has focused on special cases of CRNs with special simplifying features in their interaction structures, including weakly reversible (WR) and deficiency zero (DZ) networks [72]. However, large and complex CRNs found in real-world biological systems rarely fall into these special categories [73]. In particular, the large CRN we present here for the regulation of cholesterol in the retina eludes such simple categories. Nevertheless, a directed graph structure representing the CRN can be constructed, organizing the reactions into connected components (known as ‘linkage classes’ in chemical reaction network theory [72]) and then decomposed into algebraically independent subnetworks (see Supplementary Information of [19] for a detailed discussion of this rationale). By partitioning the CRN reactions into algebraically independent (i.e. rank additive) subsets, the steady states of the molecules within the associated subnetworks can be determined independently from their complement in the CRN. Importantly, these independent subnetworks allow discernment between network reactions that contribute to the network’s RPA capacity and network reactions that play no role in the RPA capacity of the CRN [19,21].

**Figure 4.**
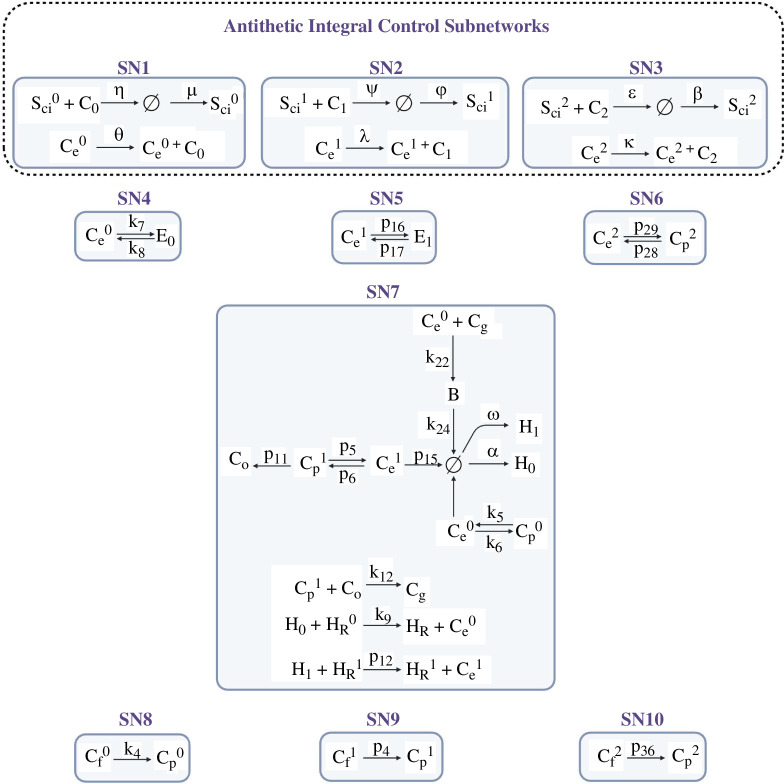
Independent subnetworks responsible for imposing RPA on cholesterol concentrations in the human retina. (Created with BioRender.com.)

Here we provide open-source code to accomplish the systematic decomposition of the retinal cholesterol CRN into the finest partition of independent subnetworks (see figure 4). This new code adapts the Matlab function developed in [73] into a freely available Julia implementation which additionally exploits the functionality of the symbolic modelling Julia package, Catalyst. Data and relevant code for this research work are stored in GitHub: https://github.com/noa-l-levi/Independent_decompositions_with_catalyst and have been archived within the Zenodo repository: https://doi.org/10.5281/zenodo.13328876.

Figure 4 depicts the 10 independent subnetworks that are responsible for conferring RPA on the species given in table 4, as revealed by our Julia code, providing a set of independent graph structures that can confirm the RPA capacity of these species. All remaining reactions (i.e. those not featured in figure 4) are independent of the depicted reactions, and therefore do not contribute to the homeostatic regulation of cholesterol in this network.

As shown in figure 4, our analysis reveals the remarkable finding that there exist three independent antithetic integral controllers in the CRN (SN1, SN2 and SN3), each imposing RPA on $C_{e}^{0}$ , $C_{e}^{1}$ and $C_{e}^{2}$ , respectively. Each of these independent subnetworks has a deficiency of one, and imposes RPA on the noted species by the Shinar–Feinberg theorem [74] due to the presence of the ‘null’ complex ( ⌀ ) in the associated subnetwork. (We refer interested readers to a detailed discussion of these graph–theoretic technicalities in the Supplementary Information of [19].) Thus, the molecules $C_{e}^{0}$ , $C_{e}^{1}$ and $C_{e}^{2}$ are under the direct control of the noted RPA-conferring mechanism.

By contrast, the remaining RPA-capable molecules inherit the RPA property from $C_{e}^{0}$ , $C_{e}^{1}$ and $C_{e}^{2}$ through their topological relationships to these three, via additional independent graph structures (SN4 to SN10 in figure 4). In particular, SN4, SN5 and SN6 are all deficiency zero, and comprise a single terminal strong linkage class, allowing $E_{0}$ to inherit the RPA property from $C_{e}^{0}$ , $E_{1}$ from $C_{e}^{1}$ and $C_{p}^{2}$ from $C_{e}^{2}$ . SN7 has a deficiency of three. The mass–action equations derived from this independent subset can be analysed using our above-noted Singular code, which confirms that the RPA property is transferred to the molecules $C_{p}^{0}$ , $C_{p}^{1}$ , $C_{O}$ , $C_{g}$ and B from their graph relationships to $C_{e}^{0}$ and $C_{e}^{1}$ . Finally, the independent subnetworks SN8, SN9 and S10 allow the transfer of RPA from $C_{p}^{0}$ to $C_{f}^{0}$ , $C_{p}^{1}$ to $C_{f}^{1}$ and $C_{p}^{2}$ to $C_{f}^{2}$ . The RPA-transferring action of each of these subnetworks is independent of the actions of all other subnetworks of the CRN.

**Figure 5.**
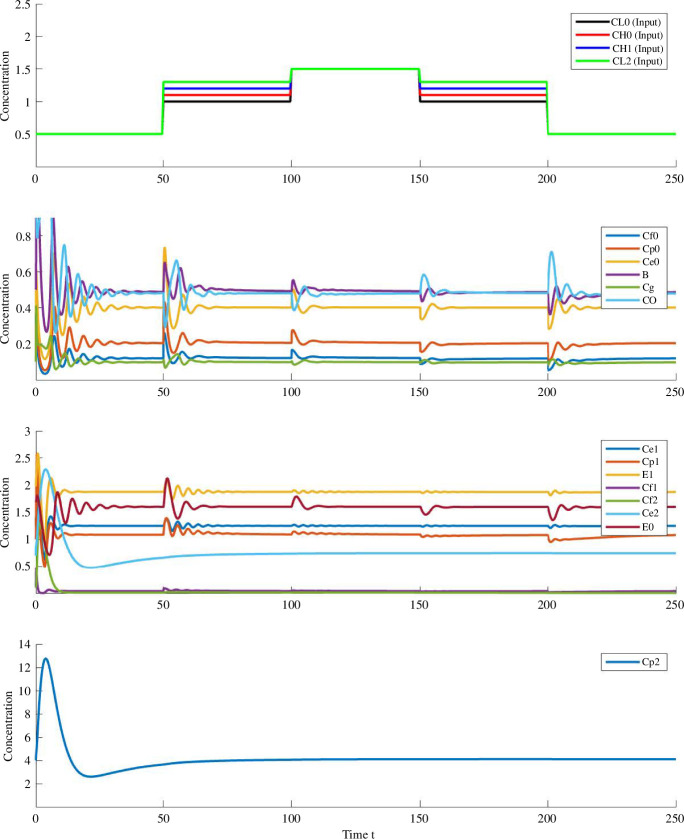
Numerical simulation of equations (3.1)–(3.41) in response to the noted step changes in $C_{L}^{0},C_{H}^{0},C_{H}^{1}$ and $C_{L}^{2}$ . Only species exhibiting RPA are depicted here.

We confirm the RPA property for all above-mentioned RPA-capable molecules (as listed in table 4) through numerical simulations of equations (3.1)–(3.41) using a range of step functions for the input molecules ( $C_{L}^{0},C_{H}^{0},C_{H}^{1}$ and $C_{L}^{2}$ ), and a range of different parameter sets. We provide a representative example in figure 5 which illustrates the return to the expected setpoint for each RPA-capable molecule. Parameters and initial conditions for the solutions depicted in figure 5 are:


$$p_{1}=0.5,p_{2}=16,p_{3}=15,p_{4}=20,p_{5}=3,p_{6}=2.8,p_{7}=p_{9}=p_{13}=p_{15}=p_{18}=p_{19}=p_{26}=p_{31}=p_{32}=p_{35}=1,p_{8}=p_{12}=p_{20}=p_{25}=p_{28}=p_{37}=p_{38}=10,p_{10}=3,p_{11}=0.9,p_{16}=30,p_{17}=20,p_{21}=11,p_{29}=55,p_{33}=20,p_{34}=0.7,p_{36}=p_{39}=2,k_{1}=2,k_{2}=3,k_{3}=k_{8}=k_{14}=k_{20}=0.5,k_{4}=60,k_{5}=50,k_{6}=10,k_{7}=2,k_{9}=2,k_{10}=1.2,k_{11}=k_{27}=1,k_{12}=k_{19}=10,k_{13}=0.1,k_{15}=300,k_{16}=46,k_{17}=40,k_{18}=20,k_{21}=18,k_{22}=25,k_{24}=2,k_{26}=3,k_{28}=40,k_{29}=5,\mu =2,\eta =10,\alpha =2,\phi =50,\lambda =40,\omega =1.5,\kappa =20,\psi =13,\epsilon =15,\delta =5,\theta =5,\beta =10,S_{ci}^{0}=S_{ci}^{1}=5,C_{0}=C_{1}=C_{2}=10,S_{r}^{0}=S_{h}^{0}=R_{0}=H_{R}^{0}=H_{0}=S_{p}^{0}=P_{0}=S_{b}^{0}=R_{b}^{1}=H_{R}^{1}=C_{O}=C_{g}=B=S_{r}^{2}=S_{h}^{2}=S_{p}^{2}=R_{2}=P_{2}=C_{f}^{2}=C_{e}^{2}=H_{2}=H_{R}^{2}=1,S_{h}^{0}=2,C_{f}^{0}=C_{p}^{0}=50,C_{e}^{0}=C_{e}^{1}=10,E^{0}=E_{1}=5,S_{p}^{0}=0.1,R_{b}^{0}=25,C_{f}^{1}=1,S_{b}^{1}=30,S_{h}^{1}=1,C_{p}^{1}=40,C_{p}^{2}=10).$$

In summary, while modelling the reaction rates of the CRN, we appealed to the assumption of mass–action kinetics, which means that each reaction proceeds at a rate in direct proportion to the concentrations of its reactants. The utility of the mass–action assumption for the analysis of the model as a whole is that it produces polynomial equations, which may be analysed easily using Gröbner basis methods (as shown in our provided open-source code). Our analysis by this technique reveals that a linear coordinate change is sufficient to identify the all-important RPA-invariants for retinal cholesterol concentrations; as such, reaction kinetics can be altered post-hoc, since the functional forms of individual reaction kinetics comprise a basis for the vector space of biochemical reaction rates, which are preserved by linear transformations. Indeed, we emphasize that the identification of the RPA-capacity of $C_{e}^{0},C_{e}^{1}$ and $C_{e}^{2}$ is a consequence only of the graph structure of the chemical reaction network, and its decomposition into independent subsets, as shown in figure 4. Thus, our conclusions as to the RPA capacity of the noted cholesterol concentrations are independent of any assumptions on reaction kinetics, and highlight the fact that RPA is conferred directly to the respective $C_{e}$ concentrations by the ‘antithetic’ interactions involving the SREBP/Scap/Insig complexes ( $S_{ci}^{i}$ ) and the ‘active’ cholesterol ( $C^{i}$ )—see Subnetworks SN1, SN2 and SN3, figure 4. In addition, the graph relationships of other RPA-capable forms of cholesterol transfer the RPA property from $C_{e}^{i}$ to these molecules (see Subnetworks SN4–10, figure 4), guaranteeing a kinetics-independent form of RPA in all cases.

## 5. Discussion

Within the vertebrate retina, cholesterol is believed to exhibit a stringent form of homeostatic regulation, and thus robust perfect adaptation (RPA), which is of central importance to ensuring its proper structure and function. However, the precise molecular mechanisms underlying such robust homeostatic control have remained elusive until now. This lack of fundamental understanding of retinal cholesterol homeostasis may be attributed primarily to the complex and intricate nature of the signalling network governing cholesterol regulation, involving several dozen distinct molecular forms, compared with the relative simplicity of most documented RPA-capable CRNs. But recent technical developments in the mathematical study of universal RPA-capable CRN structures [18,19,70,71,75,76] have now opened up new opportunities to understand homeostatic control in complex networks such as the multi-compartment tissue-level regulation of cholesterol concentration of the human retina.

Our previous analysis of cholesterol regulation at the single-cell level [21] demonstrated that an antithetic integral controller is responsible for the tight homeostatic control of the concentration of intracellular cholesterol. Here, by contrast, our multi-compartment CRN representation of cholesterol regulation within the human retina, involving three connected tissue compartments of the RPE, a Müller cell layer and a photoreceptor layer, reveals a more complex picture of the homeostatic control of cholesterol in this tissue-level setting. Nevertheless, one core feature is preserved from the simpler cholesterol regulatory CRN at the cellular level: an antithetic integral controller structure remains the driver mechanism for cholesterol homeostasis in several molecular forms, even in this highly complex multi-compartment CRN.

Thus, for cells in each of the distinct tissue compartments of the retina, a very specific controller structure tightly regulates the concentration of cholesterol in that compartment—independently of the network structures in other tissue compartments and in spite of the network interconnections among the compartments. As a consequence, there is no single homeostatic setpoint for cholesterol that is orchestrated for the entire tissue. Indeed, we show that the large and complex regulatory network encompassing three different tissue compartments (involving transfer of cholesterol among compartments as well as perturbations to various forms of cholesterol concentration via delivery from the circulation) decomposes into a modular structure, each module being characterized by the specific and well-defined network topology known as an opposer module. Each such module imposes direct control over endoplasmic reticulum (ER) cholesterol in the compartment it regulates through a well-defined antithetic integral control mechanism involving the high-affinity binding of the SREBP/Scap/Insig complex to active cholesterol. As a consequence, the tightly regulated concentration of ER cholesterol in a particular compartment has its own independent setpoint, determined by the constitutive production rate of SREBP/Scap/Insig and the release rate of cholesterol into the ER-lumen in that compartment. In other words, there are three antithetic integral controllers at work to maintain retinal cholesterol homeostasis, and each of these operates independently of the other two.

We observe that the RPA-promoting interactions between the SCREBP/Scap/Insig complex and active cholesterol are mathematically identical to the nature of the interactions between, say, sigma/anti-sigma factors in bacteria [20,21], and to antithetic interactions that implement RPA in synthetic biology studies [20]. These antithetic interactions are entirely distinct from the zero-order integral-computing interactions, involving enzymes, that have been identified as a source of RPA-conferring integral control in many other types of biochemical networks [75–77].

From a topological viewpoint, our network analysis suggests that retinal cholesterol regulation relies upon three independent Opposer modules, and not a three-node opposing set [18,19,70,71]. From the tightly regulated ER cholesterol in each retinal compartment, RPA is then imposed indirectly, via backpropagation along the associated opposer module’s feedback structure, on a variety of other cholesterol forms in the same compartment. Of these RPA transferring mechanisms, the RPE contains the most complex RPA-conferring substructure: a deficiency-three subnetwork which transmit the RPA property to the ApoB-lipoprotein particle ( B ). It is thus interesting to note that our model analysis is in qualitative agreement with established biological studies since this is precisely the molecule suggested by Curcio *et al*. [9] to be the key contributor of cholesterol accumulation in drusen—an important driver in the development of macular degeneration. Furthermore, RPA of the ApoB-lipoprotein particle concentration at the setpoint indicated in table 4 agrees with the observation from the clinical biology literature that, in healthy young eyes, these formed particles are consistently removed to the circulation to maintain a steady concentration of the lipoprotein particle in BM [9–11]. It is clear from our model that the setpoint of the ApoB-lipoprotein particle concentration is a function of many different zero-order synthesis rates, which could certainly be subject to variability, particularly in an ageing eye or under the conditions of a disease-state. From this standpoint, our modelling is consistent with the published biological data that indicates a slow build-up of individual lipoprotein particles over time in the ageing eye [9,11].

Indeed, homeostasis of the ApoB-lipoprotein particle is regulated by a more complex mechanism than antithetic integral control and is a consequence of a ‘backpropagation’ process within the feedback structure of an RPA opposer module [18,19]. This newly identified homeostatic mechanism may now offer insights to the scientific community in examining potential mechanisms for perturbation to this robust cholesterol homeostasis in the context of diseases such as age-related macular degeneration (AMD). Furthermore, the rate constants involved in conferring RPA to the ER cholesterol point to the involvement of the SREBP complex and the active cholesterol in the ER.

The mathematical model we present here makes clear that the constitutive production of the SREBP/SCAP/Insig complex ( $S_{ci}$ ), as well as the sensing of active cholesterol molecules ( $C_{i}$ ) by this complex, is fundamental to the robust homeostatic regulation of cholesterol at the cellular level and ultimately throughout the retina. To our knowledge, no mathematical models exist that describe the mechanisms by which the SREBP/SCAP/Insig complex implements integral control to maintain cholesterol homeostasis in the cellular layers of the retina. The mechanisms implemented in our model not only replicate published observations that the SREBP/SCAP/Insig complex acts as part of a feedback regulator for cholesterol homeostasis at the cellular level but further identify its contribution to integral control, and thus, RPA.

We acknowledge that while the primary signalling pathways influenced by SREBPs have been thoroughly mapped out, the mechanisms regulating SREBPs themselves are notably intricate and complex. In building our theoretical model, we included those features of our system thought to be significant and of relevance to the question under consideration. Specifically, we assumed that it is through the sensing of cholesterol by Scap that the transport of the SREBP/Scap complex to the Golgi is inhibited. It is, however, also hypothesized that the ER membrane protein known as ring finger protein 139 (TRC8) could inhibit the processing of SREBP2 through sterol sensing. In a similar way, the TRC8 protein forms a complex with SREBP2 and SCAP, which prevents SCAP from binding to COPII proteins and inhibits the transport of the SREBP/SCAP complex to the Golgi apparatus [78]. The inhibitory effect of TRC8 on cholesterol metabolism aligns with its proposed role as a tumour suppressor, given that tumour cells require substantial amounts of membrane cholesterol for their rapid growth. While we have not incorporated this alternative SREBP/SCAP/TRC8 complex formation in our modelling, our results are nevertheless consistent with the current state of knowledge as described in the seminal studies by Goldstein *et al*. [54].

Of the main sterol regulatory-element binding proteins, SREBP2 regulates the transcription of genes involved in cholesterol metabolism. A recent review by Shimano and Sato [79] points to several studies that have investigated the pathophysiological influence of SREBP2 at the cell, tissue, organs and organism level. Having established that RPA is achieved in the three connected tissue compartments in the retina, we offer our detailed mathematical model as a framework that could be tested experimentally and developed further in future work. For example, it is hypothesized that the up-regulation of cholesterol biosynthesis in many cancers may be due to mutations in the gene transcription of SREBP2 [80], while it has also been shown that the mammalian target of rapamycin complex 2 protein Kinase B (TORC2-AKT) signalling may cancel the activation of SREBPs. This dysregulation might contribute to insulin resistance and diabetes mellitus [81]. Furthermore, when SREBP2 is overexpressed in pancreatic β -cells of mice, it induces β -cell death, thereby disrupting glucose tolerance and potentially leading to diabetes mellitus Ishikawa 2008. Given SREBP2’s role in cholesterol synthesis, novel therapeutic strategies targeting it hold promise for treating cancers with abnormal cholesterol metabolism [82]. While evidence exists for the effects of disrupted SREBP2-regulated cholesterol metabolism in vertebrate and mammalian cells [79], to our knowledge, no similar studies on the impact of SREBP2 disruption in mammalian retinal cells exist currently.

## Data Availability

Data and relevant code for this research work are stored in GitHub and have been archived within the Zenodo repositories [83,84].
